# Alleviating calf weaning stress: the important role of maternal bovine appeasing substance in calf husbandry management

**DOI:** 10.5713/ab.250567

**Published:** 2025-12-18

**Authors:** Tian-Lin Zhang, Jie Wang, Jie Sun, Bo-Shi Yan, Lin Wang

**Affiliations:** 1 College of Veterinary Medicine, Shandong Provincial Key Laboratory of Zoonoses, Shandong Agricultural University, Tai’an, China; 2 Rencheng District Government Service Center, Jining, China

**Keywords:** Calf, Growth Performance, Health, Immunity, Inflammation, Maternal Bovine Appeasement Substances, Weaning Stress

## Abstract

**Objective:**

Weaning stress impairs calf health by causing growth retardation and immune dysfunction, underscoring the need for targeted interventions. Maternal bovine appeasing substance (MBAS) has been proposed as a novel therapy for stress in cattle, but its efficacy against weaning stress remains unclear. This study assessed the health impacts of weaning and evaluated MBAS as a potential intervention.

**Methods:**

Health impacts were first evaluated in 60 weaned Holstein calves. Then, another 60 healthy calves of similar age were randomly divided into two groups (n = 30/group). Control calves received a single 2.5 mL saline spray one day pre-weaning, while the MBAS group received a single 2.5 mL spray of MBAS (100 mg/mL).

**Results:**

Weaning stress increased diarrhea (56.52% to 78.26%) and respiratory disease incidence (10.17% to 15.88%), elevated exercise steps (32,196±1,267 vs 38,226±1,132, p = 0.0043), and reduced dry matter intake (2.78±0.03 vs 2.59±0.06 kg/d at D4, p = 0.0041) and average daily gain (ADG, 0.89±0.05 vs 0.71±0.04 kg/d, p = 0.0230) within 15 days post-weaning. MBAS improved ADG (0.92±0.15 vs 1.25±0.14 kg/d at D7, p = 0.0120), lowered respiratory disease (23.07% to 17.42%) and diarrhea incidence (83.70% to 52.70%), and shortened diarrhea duration (4.32±0.47 d vs 3.15±0.32 d, p = 0.0090). It also reduced serum interleukin-1 (49.39±2.40 vs 43.51±1.64 pg/mL, p = 0.0350), haptoglobin (58.29±3.3 vs 49.40±3.65 μg/mL, p = 0.0050), and cortisol (37.42±1.67 vs 33.05±2.24 nmol/L, p = 0.0310), while increasing immunoglobulin G (10.30±1.46 vs 13.50±1.58 μg/mL, p = 0.0131) and interferon-γ (6.89±0.47 vs 7.94±0.63 ng/L, p = 0.0330) on D5.

**Conclusion:**

MBAS alleviates weaning stress by inhibiting inflammation, enhancing immunity, and reducing anxiety, providing an effective strategy to improve calf health.

## INTRODUCTION

Calves are usually exposed to several stressors throughout their growth phase, including transportation, disbudding/castration, weaning, and commingling [1,2]. Most of these stressors would appear to be unavoidable in a calf-feeding production system. Among these stressors, weaning is an important one affecting calf health [3]. It seems to be beneficial to consider doubling the birth weight as the start time for weaning, which is cited as a standard practice for weaning calves [4]. However, this practice may not always be appropriate for calf health and growth during certain critical windows in the calf’s life [5]. The average weaning time for most calves is 2 months of age, which is 6 to 10 months earlier than natural weaning of calves [6]. Thus, during weaning, calves enter a period of high stress and face many physiological challenges that directly affect their growth performance, health, and welfare [7].

Calves are highly susceptible to intestinal disease during the first or second week of birth, with about 1 in 3 calves suffering from intestinal disease [8]. The risk of intestinal disease is greatest at 1 week of age, and the risk of respiratory disease steadily increases and stabilizes after 4 weeks of age [2]. The weaned calves are more susceptible to intestinal and respiratory diseases due to the immune system still adapting and developing [9]. Furthermore, weaning stress stimulates an acute phase inflammatory response with immediate and long-term effects on calf growth and immunity [10]. A previous study showed that calves with higher body weight coped more effectively with stress compared to calves with low body weight [11]. Thus, it is important to find measures to improve the performance and health of weanling calves by alleviating stress-induced adverse responses.

Appeasing substance is an effective chemical for alleviating stress response in ruminants and was originally used for reducing stress behaviors in piglets [9]. The appeasing substance is generally considered to exert an anti-stress effect by stimulating the nasal plough in the nasal cavity [12], which in turn affects and regulates the neurological and hormonal secretion system of the hypothalamus [13]. In recent years, maternal bovine appeasing substance (MBAS) has been used to alleviate multiple factors induced stress in dairy and beef cattle [14,15], which improves animal welfare and reduces farm losses.

While previous studies have demonstrated the efficacy of MBAS in alleviating stress in beef and dairy cattle during various stressful events such as castration, feedlot entry, and transport [9,16], its targeted application and comprehensive evaluation specifically for dairy calf weaning stress remains to be elucidated. Most existing research has focused on composite stressors or older animals. Therefore, this study was designed to systematically evaluate the efficacy of a single pre-weaning MBAS application on a comprehensive set of parameters, including growth performance, detailed disease incidence and duration, behavioral activity, and a panel of serum stress, inflammatory, and immune biomarkers in dairy calves, thereby providing a more holistic understanding of its role in managing this critical developmental challenge.

## MATERIALS AND METHODS

### Animals and experimental design

This experiment was conducted in a large modern intensive dairy farm (Tangshan, Hebei Province, China). This study first investigated the epidemiology of weaning stress in 60 Holstein calves, covering a period from 10 days pre-weaning to 15 days post-weaning. The calves’ average age and body weight were 57.5±0.5 days and 85.5±3.2 kg. Then, 60 healthy Holstein calves were reselected for the MBAS experiment based on similar body condition and age from those born subsequently. The calves’ average age and body weight were 58.5±0.5 days and 86.7±2.8 kg. 60 calves were randomly divided into two groups (n = 30/group): the control group (received a single 2.5 mL spray of saline on the skin surfaces of the head and nose) and the MBAS group (received a single 2.5 mL spray of MBAS at the same location). The administration was only performed once at 09:00 am on the day before weaning (1 d pre-weaning). MBAS (100 mg/mL) was purchased from FERA Diagnostics and Biologicals Corp (FerAppease). The dosage and administration protocol (a single 2.5 mL spray) were selected based on the manufacturer’s recommendations. The 2.5 mL volume is sufficient to cover the calf’s nasal planum and surrounding area upon application, thereby preventing inadequate coverage from an insufficient amount, while also avoiding liquid waste and potential animal discomfort that could result from an excessive volume. The experiment lasted from 1 day pre-weaning to 15 days post-weaning. Throughout the study, which included the pre-weaning period, all calves were individually housed in hutches or pens as per standard farm management. This housing arrangement allowed for the accurate measurement of individual feed intake, health status, and activity levels. The calves were transitioned to *ad libitum* feeding once daily feed intake consistently exceeded 1.5 kg for three consecutive days and body weight reached approximately twice their birth weight. The feed ratios and feeding standards for the calves are presented in Tables 1, 2.

### Incidence of diarrhea

Feces of calves were observed daily from 08:00–10:00 and 15:00–17:00 during the period spanning 15 days pre weaning to 15 days post-weaning. The number of diarrheic calves and dates of onset were recorded using standardized diagnostic criteria (Table 3), which refine established methods [17–19] with greater granularity. Feces were cleared daily at 17:00 to ensure consistent observation conditions. Diarrhea incidence was calculated as: Diarrhea incidence (%) = (Number of diarrheic calves / Total calves at risk)×100%.

### Incidence of respiratory diseases

Professional veterinarians monitored calf respiratory health status daily from 15 days pre-weaning to 15 days post-weaning, with observations conducted between 08:00–10:00 and 15:00–17:00. The number of affected calves and dates of respiratory disease onset were recorded using standardized diagnostic criteria (Table 4). These criteria integrate established methods from prior studies [20–22], adapted to our feeding environment to account for variations in sensitivity and specificity across scoring systems. Respiratory disease incidence was calculated as: Respiratory disease incidence (%) = (Number of diarrheic calves / Total calves at risk)×100%.

### Average intake of dry matter

The total weight of pellets added to the calf feed bowl was weighed and recorded at 07:00 daily using an electronic balance, and the total residual pellets were weighed and recorded at 07:00 on the following day. During that time, the feed bowl is installed 60 cm above the ground and tilted inward at a 15-degree angle to prevent contamination from urine and feces. Furthermore, during inspections every 2 h, contaminated or spilled feed is immediately removed and not included in the residual weight (recorded as waste). Dry matter intake (DMI) calculations account for all losses: DMI (kg/day) = (Total feed added − [Total clean residue + waste] / Number of calves). Waste (Contaminated + Spilled feed) averages less than 2% of the feed provided, ensuring comparable exposure between groups.

### Average number of exercise steps

The ear tag was placed between the two cartilages of the calf’s right ear at 1 month of age, as close to the head as possible, and avoiding blood vessels to prevent the tag from falling out. The ear tags were soaked in 75% alcohol for 30 min before use, and the area where the ear tags were placed was also sterilized with 75% alcohol to prevent infection. After ear tagging, the ear tag gateway was fixed within 50 m of the tagged animal to facilitate signal reception. The calves’ exercise steps were recorded at 22:00 by logging on the Smart Ear Marker Platform (http://lora.yimaiiot.com:59000/index, Zhongnong Zhilian Technology) each day. The difference in step count between the cumulative steps at 22:00 on the current day and the cumulative steps at 22:00 on the previous day represented steps taken during the 24 h ending at 22:00 each day.

### Average daily weight gain

Calf body weights were measured using a calibrated weighing scale at three time points: 15 days pre-weaning (D–15), at weaning (D0), and 15 days post-weaning (D15). Average daily weight gain (kg/d) = (Final weight [kg] − Initial weight [kg])/15 (d).

### Collection of blood samples

All blood samples of calves were obtained from the jugular vein and placed in vacuum tubes without anticoagulant at 10:00 am on D–1 (1 day pre-weaning), D1 (1 day post-weaning), D3, D5, D7_,_ and D14. Serum samples were obtained by centrifuging the blood samples at 1,500×g for 12 min, then stored at −20°C for later biochemical analysis.

### Analysis of serum levels of biochemical markers

Serum concentrations of immunoglobulin G (IgG), immunoglobulin M (IgM), interleukin-1β (IL-1β), interferon-γ (IFN-γ), and haptoglobin were quantified using ELISA kits (Qiyi Biological) according to the manufacturer’s protocol. Standard curves were generated from 7-point serial dilutions. Aliquots of 10 μL serum (diluted as specified in the kit instructions) were added to each well, followed by 60 min incubation at 37°C in the dark. After five wash cycles, horseradish peroxidase (HRP)-conjugated secondary antibody was added and incubated for 30 min before reaction termination. Serum cortisol levels were measured using kits from Genmei Biotechnology. Optical density values were read on a BioTek Synergy H1 microplate reader.

### Statistical analysis

All statistical analyses were performed using SPSS 27.0 (IBM). Continuous data are expressed as mean±standard error of the mean (SEM). A significance level of * p<0.05 was considered statistically significant, and ** p<0.01 was considered highly significant. For continuous data with repeated measures (e.g., daily exercise steps, DMI, and serum biomarkers collected over multiple time points), a linear mixed-effects model was employed. The model included Treatment (Control or MBAS), Time (day of measurement), and their Interaction (Treatment×Time) as fixed effects. Calf ID was included as a random effect (random intercept) to account for the correlation between repeated measurements from the same animal. For the covariance structure of the within-calf errors, a first-order autoregressive structure [AR(1)] was selected where appropriate, based on model fit criteria (Akaike’s information criterion). Between-group differences at specific time points were estimated using estimated marginal means with Tukey-Kramer adjustment for multiple comparisons. For non-repeated endpoints (e.g., total average daily gain over the 15-day post-weaning period, final body weight), data were analyzed using a linear model. This model was specified with Treatment as a fixed effect and Calf ID as a random effect, making it conceptually consistent with the mixed model used for repeated measures, but without the repeated measure of time or the time and interaction fixed effects.

## RESULTS

### Weaning stress elevated the incidence of diarrhea and respiratory diseases in calves

**Figure 1:**
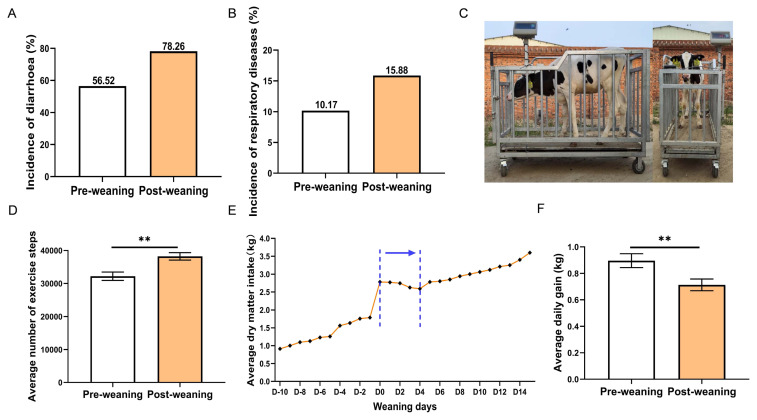
Impact of weaning stress on diarrhea incidence, respiratory disease occurrence, and average daily gain in calves. (A) Incidence of diarrhea in calves pre- and post-weaning (n = 60). (B) Incidence of respiratory diseases in calves pre- and post-weaning (n = 60). (C) The electronic scale is used to measure the weight of calves. (D) Average daily weight gain of calves pre- and post-weaning (n = 60). (E) Average dry matter intake of calves pre- and post-weaning (n = 60). Dashed blue lines indicate a transient decrease in feed intake after weaning (D0–D4). (F) Average number of steps taken by calves pre- and post-weaning (n = 60). Data represent mean±SEM. ** p<0.01. SEM, standard error of the mean.

Epidemiological survey data showed that the incidence of diarrhea and respiratory diseases in weaned calves increased by 21.74% and 5.71%, respectively, during the first 15 days post-weaning compared to the pre-weaning period (Figures 1A, 1B). The weighing results revealed that the daily weight gain of calves post-weaning (0.71 kg/day) was lower than that pre-weaning (0.89 kg/day) (Figures 1C, 1D). Correspondingly, the calf’s DMI gradually increased pre-weaning, especially on the day of weaning. However, the DMI decreased continuously from 0 to 4 days post-weaning until it started to increase at 5 days post-weaning (Figure 1E). Furthermore, the exercise steps of weaned calves were significantly increased compared to pre-weaning (Figure 1F, p<0.01).

### Maternal bovine appeasing substance attenuated weaning stress-induced growth suppression in calves

**Figure 2:**
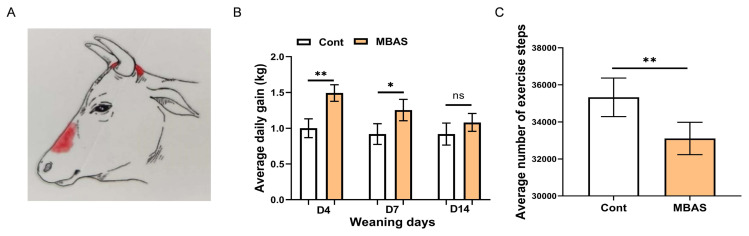
Effect of MBAS on average daily weight gain and exercise steps of weaned calves. (A) Schematic of the location where calves were coated with MBAS. (B) Average daily weight gain of weaned calves (n = 30). (C) Average number of locomotor steps of weaned calves (n = 30). Data represent mean±SEM. * p<0.05, ** p<0.01. MBAS, maternal bovine appeasing substance; SEM, standard error of the mean.

As shown in Figure 2A, MBAS was administered via spray to specific areas of calves 1 d pre-weaning. The results showed that the daily weight gain of calves in the MBAS group was significantly higher than the control group at 4 d and 7 d post-weaning (Figure 2B). Although the daily weight gain in the MBAS group showed a tendency to be higher than in the control at 14 days after weaning, the difference was not statistically significant (p>0.05). Furthermore, MBAS significantly reduced exercise steps of calves compared to the control group during 15 days post-weaning (Figure 2C, p<0.01). Based on the above results, it can be concluded that MBAS mitigated the decline in calf growth performance induced by weaning stress.

### Maternal bovine appeasing substance reduced weaning stress-induced diarrhea and respiratory disease in calves

**Figure 3:**
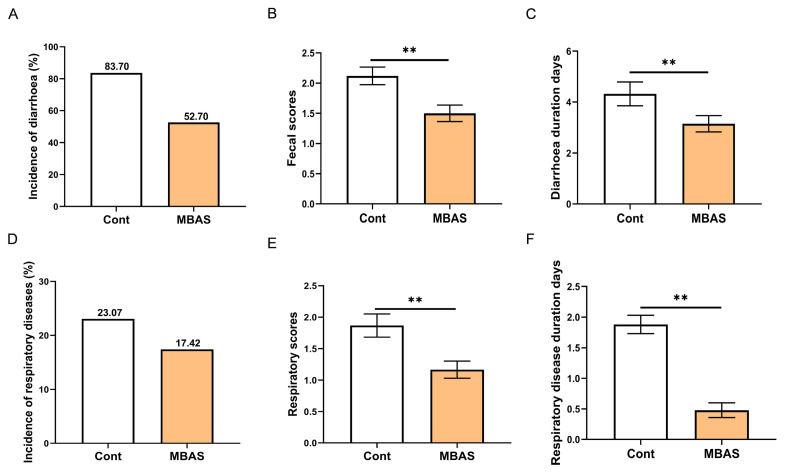
Effect of MBAS on the incidence and duration of diarrhea and respiratory diseases in weaned calves. (A) Incidence of diarrhea in weaned calves (n = 30). (B) Fecal scores of calves 15 days post-weaning (n = 30). (C) Duration of diarrhea in weaned calves. (D) Incidence of respiratory disease in weaned calves (n = 30). (E) Respiratory scores of calves 15 days post-weaning (n = 30). (F) Duration of respiratory disease in weaned calves. Data represent mean±SEM. ** p<001. MBAS, maternal bovine appeasing substance; SEM, standard error of the mean.

The incidence of diarrhea in calves was elevated across all severity levels during the 15 days after weaning compared to the 10 days before weaning (Table 5). In addition, MBAS-treated calves exhibited significantly lower fecal scores and a reduced incidence of diarrhea relative to the control group within the 15-day post-weaning period (Figures 3A, 3B). More importantly, in calves that developed diarrhea, the MBAS treatment significantly shortened the duration of diarrhea (Figure 3C). In addition, the research results showed that both the incidence rate of respiratory diseases and the respiratory system scores of the calves in the MBAS group were significantly lower than those in the control group (Figures 3D, 3E). Meanwhile, the MBAS treatment also significantly reduced the duration of respiratory diseases in calves (Figure 3F, p<0.01).

### Maternal bovine appeasing substance restored weaning stress-suppressed immunity in calves

**Figure 4:**
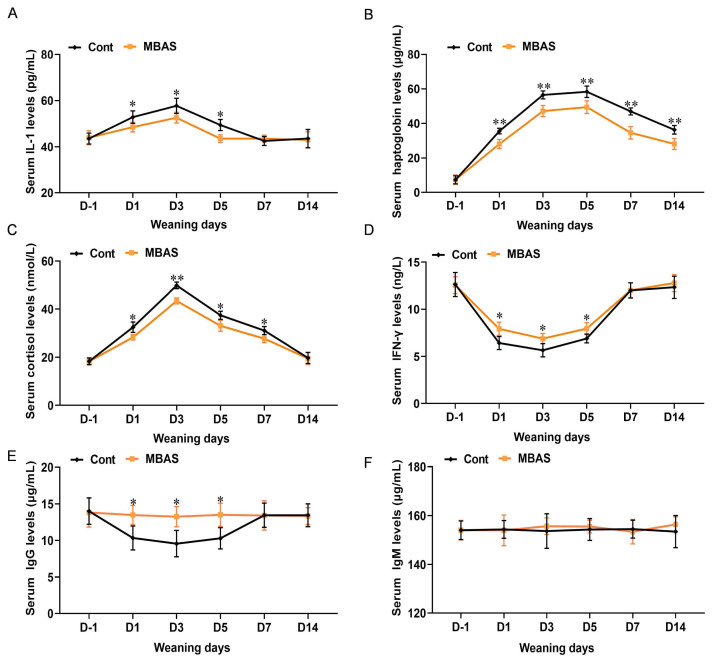
Effect of MBAS on serum stress and immune biomarkers in weaned calves. (A) Levels of IL-1 in serum of weaned calves (n = 30). (B) Levels of haptoglobin in serum of weaned calves (n = 30). (C) Levels of cortisol in serum of weaned calves (n = 30). (D) Levels of IFN-γ in serum of weaned calves (n = 30). (E, F) Levels of IgG and IgM in serum of weaned calves (n = 30). Data represent mean±SEM. * p<0.05, ** p<0.01. MBAS, maternal bovine appeasing substance; IL-1, interleukin-1; IFN-γ, interferon-γ; IgG, immunoglobulin G; IgM, immunoglobulin M; SEM, standard error of the mean.

This study investigated the effects of MBAS on inflammatory indices in weaned calves. The results showed that prolonged weaning duration progressively increased serum levels of IL-1, haptoglobin, and cortisol in calves (Figures 4A–4C, p<0.01), while concentrations of IFN-γ and IgG significantly declined within the first week post-weaning (Figures 4D, 4E, p<0.01). MBAS treatment effectively counteracted these dynamics across matched timepoints, attenuating the rise in IL-1, haptoglobin, and cortisol and mitigating the reduction in IFN-γ and IgG. Notably, serum IgM levels remained comparable between groups (Figure 4F, p>0.05), whereas cortisol concentrations were consistently lower in MBAS-treated calves than in controls throughout the observation period.

## DISCUSSION

Weaning stress generally increases the risk of disease in calves, which significantly impacts their health, growth performance, and leads to substantial economic losses for the cattle industry [4]. In this study, weaning stress notably reduced calves’ feed intake, average daily weight gain, and body immunity while increasing the incidence of diarrhea and respiratory diseases. However, these negative effects of weaning stress on calves were effectively alleviated by MBAS. These findings indicate that using MBAS pre-weaning is an effective strategy to reduce weaning stress in calves, offering a new approach to enhance the health and growth performance of weaned calves. Furthermore, the observed treatment-by-time interaction for several key parameters underscores that the efficacy of MBAS is not static but is particularly critical in modulating the calf’s response during the immediate post-weaning period.

Diarrhea is one of the most common diseases occurring in weaned calves, which leads to dehydration [23], weight decline, and even death [24]. Meanwhile, the respiratory system of weaned calves is susceptible to infection by pathogenic microorganisms, especially Pasteurella and Mannheimia haemolytica [13], thus causes respiratory diseases. In this study, the significantly higher incidence of diarrhea and respiratory diseases in calves may be related to the decreased immunity of calves due to anxiety and restlessness during the first 15 d post-weaning. The lack of teat-suckling in weaned calves increases oral stereotyping (non-nutritive oral behaviors, cross-sucking) [25], which suppresses the innate immune response and may be exacerbated if weaning is not done correctly or too early [26]. Furthermore, the decrease in feed intake and body weight gain in weaned calves may be attributed to the adaptation process of the change in the main mode of nutrient absorption from single intestinal absorption to the involvement of rumen microbial fermentation [6,23]. In this study, the statistical model revealed a significant treatment-by-time interaction (p<0.05) for average daily gain, indicating that the beneficial effect of MBAS on growth performance was most pronounced during the acute phase of weaning stress. A similar finding was that MBAS-administered calves diagnosed with a disease had a higher average daily gain than control animals diagnosed with a disease, even similar to healthy calves [14], suggested that MBAS may be able to improve weaned calf performance by restoring body health at a faster rate after pathogen attack. In addition, the significant increase in total exercise steps post-weaning may further highlight the state of psychological anxiety in weaned calves.

The MBAS is an effective chemical in relieving the stress in dairy and beef cows [9,16]. In the present study, MBAS effectively alleviated the decreased daily weight gain and increased exercise steps of calves induced by weaning stress, suggested that MBAS may contribute to the ability of calves to adapt to pellet feed and alleviate the nervousness of calves. This is consistent with the alteration of serum cortisol levels in calves. Similarly, previous studies found that MBAS application to weaned beef calves reduced stress-induced physiological responses [27], enhanced humoral immunity [28], and promoted calves to adapt to the new management protocol and environment [29]. It is also noteworthy that MBAS not only reduced diarrhea and respiratory disease in calves, but also reduced the duration of illness, which highlighted the efficacy of MBAS in enhancing calf immunity. However, a previous study found that MBAS did not affect disease onset, but it tended to reduce the days and cost of pharmacological interventions [13]. This difference in disease incidence by MBAS administration might have been caused by differences in the duration and period of administration.

Serum IgG and IgM levels reflect the functional status of calf humoral immunity [30]. Interestingly, MBAS did not appear to affect serum IgM levels in calves, while it significantly elevated IgG levels, which may be caused by IgM production earlier than IgG but shorter in duration than IgG. Inflammation is a central component in the development of diarrheal and respiratory diseases in calves. Haptoglobin is an acute response protein that is significantly elevated in the serum when the body triggers inflammatory responses [31] or is infected with pathogenic microorganisms [32]. In this study, MBAS effectively alleviated the elevation of IL-1 and haptoglobin induced by weaning stress, suggested that MBAS may have a role in alleviating inflammation in calves. Previous study revealed that weaning stress reduced serum IFN-γ concentration by inducing hypothalamic–pituitary–adrenal (HPA) axis response [33], which is consistent with the results of the present study. Thus, we speculate that MBAS may also increase serum IFN-γ concentration by affecting the HPA axis. When animals perceive stress, it might lead to the inflammatory cascade response that transfers nutrients from an anabolic to a catabolic state [34,35]. Dramatic increases in cortisol concentrations in the blood usually trigger an acute and temporary inflammatory cascade [13]. Furthermore, the expressions of pro-inflammatory-related factors in serum were reduced in animals receiving MBAS post-weaning and entry into the feedlot [36], further showed that inflammatory modulation might be one of the potential mechanisms underlying the observed benefits. It is noteworthy that the MBAS intervention in this study was administered as a single dose before weaning. This pre-emptive strategy was designed to ‘prime’ the neuroendocrine system ahead of the predictable stressor, and our results demonstrate its efficacy in mitigating the acute phase of the weaning response. Future research should explore the potential benefits of alternative application regimens. For instance, multiple applications encompassing the pre- and post-weaning periods might offer enhanced protection for calves facing consecutive stressors (e.g., weaning followed by commingling or transport) or could potentially extend the window of improved resilience, thereby influencing long-term health and productivity parameters. A comparative study of single versus multiple application protocols would provide valuable insights for optimizing this intervention under diverse commercial farming conditions.

## CONCLUSION

In this study, weaning stress substantially elevates the incidence of diarrhea and respiratory disease in calves while concurrently suppressing their growth performance. Critically, the MBAS application effectively attenuated several adverse effects caused by weaning stress on the calves. These findings establish MBAS as a promising intervention for enhancing health resilience and optimizing nutritional management in weaned calves, providing a translatable strategy for sustainable livestock production.

## Figures and Tables

**Table 1:** Nutritional composition of calf starters

Composition	Ratios (%)
Ingredient (% of DM)	
Ground corn	20.1
Ground wheat	14.5
Molasses	5.6
Wheat hulls	14.9
Gluten feed	10.0
Soybean hulls	8.2
Soybean meal	15.1
Corn germ meal	5.1
Copra meal	5.1
Salt	0.5
Premix^1)^	0.34
NaHCO_3_	0.5

1) The premix provides the following nutrients per kg of mixed feed: Vitamin A, 4,600 IU; Vitamin D, 769 IU; Vitamin E, 16 IU; Zinc oxide, 59 mg; MnO_2_·H_2_0, 61 mg; MgO, 1.92 g.

**Table 2:** Feeding standard of lactating calves in pasture

Days (d)	Milk	Single feeding volume (L)	Times	Daily feeding volume (L)	Days	Total volume (L)
0–1	Pasteurized colostrum	4	1	4	1	4
2–7	Pasteurized normal milk	2.5	3	7.5	6	45
8–34		3	3	9	27	243
35–50		4	3	12	16	192
51–54		2.5	3	7.5	4	30
55–57		2.5	2	5	3	15
58–65		2.5	1	2.5	8	20
66–73	Transferred to transition pen after 7 d of feeding					

**Table 3:** Diagnostic criteria for scoring calf diarrhea

Score of diarrhea	Appearance	Dry matter content (%)
0	Strips or granules	>30
1	Soft but formable faeces	25–30
2	Sticky and unformed	20–25
3	Liquid, unformed, with faecal water segregation	<20

**Table 4:** Diagnostic criteria for scoring calf respiratory diseases

Standardized scores	Cough	Nasal discharge	Eye or ear discharge
0	No	Normal, plasma	No
1	Single cough	Unilateral little, cloudy secretion	Little secretions
2	Recurrent or occasional spontaneous cough	Bilateral, cloudy or excessive mucus secretions	Bilateral secretions and slight drooping of ears
3	Recurrent spontaneous cough	Bilateral, massive, purulent mucopurulent secretions	Massive secretions, severe head tilt and drooping of both ears

**Table 5:** Frequency of diarrhea in control and MBAS-treated calves during the 10 days pre-weaning and the 15 days post-weaning

Item	Control group (%)	MBAS group (%)	p-value
Frequency of diarrhea			
Faecal score>1			
d –10–0	50.0	46.7	0.1913
d 0–15	83.7	52.7	0.0243
Faecal score = 2			
d –10–0	33.3	26.7	0.0985
d 0–15	45.7	33.7	0.0407
Faecal score = 3			
d –10–0	16.7	12.57	0.0723
d 0–15	26.7	10.0	0.0335

MBAS, maternal bovine appeasing substance.

## Data Availability

Upon reasonable request, the datasets of this study can be available from the corresponding author.
